# Efficacy and safety of oral ibrexafungerp in Chinese patients with vulvovaginal candidiasis: a phase III, randomized, double-blind study

**DOI:** 10.1007/s15010-024-02233-w

**Published:** 2024-04-03

**Authors:** Xiaoqian Wang, Wenying Wang, Jingjing Li, Ruifang An, Lihong Chen, Jiajing Lin, Dabao Xu, Jin Qiu, Weihua Song, Mijiti Patiman, Hongjie Ruan, Gang Wang, Fengxia Xue, Xu Wang, Xiaowan Luo, Qi Ruan, Ling Shi, Chun Zhang, Lina Hu, Shijin Wang, Hong Shi, Xiaoli Wang, Songling Zhang, Yingxiong Li, Jing Lu, Baojin Wang, Hongyan Xu, Hong Ye, Bei Zhang, Chunlian Zhang, Sumin Qian, Qiong Wu, Wen Jia, Chuan Li, Qinping Liao

**Affiliations:** 1 Department of Gynecology, Beijing Tsinghua Changgung Hospital, School of Clinical Medicine, Tsinghua University, Beijing, China; 2 Department of Gynecology, The First Affiliated Hospital of Xi’an Medical College, Shaanxi, China; 3 Department of Gynecology, Liuzhou Maternity and Child Healthcare Hospital, Guangxi, China; 4 Department of Gynecology, Liuzhou Hospital, Guangzhou Women and Children’s Medical Center, Guangxi, China; 5 Department of Obstetrics and Gynecology, The First Affiliated Hospital of Xi’an Jiaotong University, Shaanxi, China; 6 Department of Gynecology, Shaanxi Provincial People’s Hospital, Shaanxi, China; 7 Department of Gynecology, Liuzhou Worker’s Hospital, Guangxi, China; 8 Department of Gynecology, The Third Xiangya Hospital of Central South University, Hunan, China; 9 Department of Gynecology, Shanghai Tongren Hospital, Shanghai, China; 10 Department of Gynecology, Women & Children’s Health Care Hospital of Linyi, Shandong, China; 11 Department of Gynecology, University of Chinese Academy of Sciences Shenzhen Hospital, Guangdong, China; 12 Gynecology Department, Nanjing Maternity and Child Health Care Hospital, Jiangsu, China; 13 Department of Gynecology, Sichuan Maternal and Child Health Hospital, Sichuan, China; 14 Department of Gynecology, Tianjin Medical University General Hospital, Tianjin, China; 15 Department of Gynecology, Tonghua Central Hospital, Jilin, China; 16 Department of Gynecology, Zhongshan Women and Children’s Hospital, Guangdong, China; 17 Department of Gynecology, Shunde Women and Children’s Hospital of Guangdong Medical University, Guangdong, China; 18 Department of Gynecology, The Second Affiliated Hospital of Liaoning University of Traditional Chinese Medicine, Liaoning, China; 19 Department of Gynecology, The Central Hospital of Wuhan, Hubei, China; 20 Department of Gynecology, The Second Affiliated Hospital of Chongqing Medical University, Chongqing, China; 21 Department of Gynecology, The First Affiliated Hospital of Xinxiang Medical University, Henan, China; 22 Department of Gynecology, Dalian Medical University Affiliated First Hospital, Liaoning, China; 23 Department of Gynecology, Hainan Women and Children’s Medical Center, Hainan, China; 24 Department of Gynecology, The First Hospital of Jilin University, Jilin, China; 25 Department of Gynecology, Guangzhou Panyu Central Hospital, Guangdong, China; 26 Department of Obstetrics and Gynecology, Urumqi Maternal and Child Health Hospital, Xinjiang, China; 27 Department of Gynecology, The Third Affiliated Hospital of Zhengzhou University, Henan, China; 28 Department of Gynecology, Yuebei People’s Hospital, Guangdong, China; 29 Department of Gynecology, Yichang Central People’s Hospital, Hubei, China; 30 Department of Gynecology, Xuzhou Central Hospital, Jiangsu, China; 31 Department of Gynecology, Taihe Hospital, Affiliated Hospital of Hubei University of Medicine, Hubei, China; 32 Department of Gynecology, Cangzhou Central Hospital, Hebei, China; 33 Jiangsu Hansoh Pharmaceutical Group Co. Ltd., Jiangsu, China

**Keywords:** Vulvovaginal candidiasis, Ibrexafungerp, Candida, Antifungal, Randomized clinical trial

## Abstract

**Purpose:**

To evaluate the efficacy and safety of oral ibrexafungerp (HS-10366) versus placebo in Chinese patients with vulvovaginal candidiasis (VVC).

**Methods:**

A double-blind, placebo-controlled, randomized, multicenter phase III study was conducted in symptomatic VVC patients. Patients received (2:1) twice-daily oral ibrexafungerp 300 mg or matching placebo for 1 day. The primary endpoint was clinical cure (vulvovaginal signs and symptoms [VSS] score = 0) at test-of-cure (TOC) on day 11 ± 3. The secondary endpoints included mycological eradication, overall response, and clinical improvement (VSS score ≤ 1) at TOC, and vulvovaginal symptom resolution at follow-up on day 25 ± 4.

**Results:**

In total, 360 patients were included in the modified intention-to-treat set (defined as positive *Candida* cultured and receiving at least one study drug; 239 for ibrexafungerp, 121 for placebo). Compared with placebo, patients receiving ibrexafungerp had a significantly higher proportion of clinical cure (51.0% vs. 25.6%), mycological eradication (55.6% vs. 18.2%), overall response (33.9%, vs. 8.3%) at TOC and complete symptom resolution (74.5% vs. 39.7%, all *P* < 0.001) at follow-up. Subgroup analysis of clinical cure indicated that patients with *C. albicans* could benefit from ibrexafungerp over placebo. A similar benefit trend was also observed in those with non-*albicans Candida* by post-hoc analysis. Further analyses revealed similar efficacy of ibrexafungerp between patients with fluconazole non-susceptible *C. albicans* and fluconazole susceptible *C. albicans* regarding clinical cure and mycological eradication. Ibrexafungerp was generally well tolerated. Adverse events were primarily gastrointestinal and were mainly mild in severity.

**Conclusions:**

As a first-in-class antifungal agent, ibrexafungerp demonstrated promising efficacy and favorable safety for VVC treatment in Chinese patients.

**Chinadrugtrials.org.cn registry number:**

CTR20220918.

**Supplementary Information:**

The online version contains supplementary material available at 10.1007/s15010-024-02233-w.

## Introduction

Vulvovaginal candidiasis (VVC) is a common fungal infection caused by *Candida* species and is a source of significant morbidity in women from all social classes. [1, 2]. About 75% of women will experience at least one episode of VVC, and 40 ~ 45% will experience multiple VVC episodes [1, 3]. The most common causative pathogen is *C. albicans*, and *C. glabrata* accounts for the most non-*albicans Candida* (NAC) [2]. Currently, azole antifungals are still preferable as the first-line therapy for VVC, including oral and topical formulations. Nevertheless, long-term use of azole antifungals and abuse of over-the-counter antifungals reduced fluconazole sensitivity among *Candida* species and increased drug resistance, especially for vaginal *C. albicans* isolates in China [4–7]. And there are limited options for VVC patients with azole non-susceptible *Candida* infection or with azole intolerance. Hence, new treatment approaches and agents possessing both broad-spectrum fungicidal activity and favorable safety profile, are urgently needed.

HS-10366 (generic name: ibrexafungerp) is a first-in-class, orally active, semisynthetic, triterpenoid derivative that blocks the synthesis of the fungal cell wall polymer β-(1,3)-d-glucan, which has broad-spectrum anti-*Candida* fungicidal activity, especially against echinocandin- and azole-resistant *Candida* species[8, 9]. The efficacy and safety of ibrexafungerp for treating VVC have been evaluated in two multicenter, global, randomized, double-blind, placebo-controlled phase III clinical trials in US and Bulgaria (VANISH 303, conducted in US, NCT03734991; VANISH 306, conducted in US and Bulgaria, NCT03987620). Both of trials demonstrated the superiority of ibrexafungerp over placebo in clinical cure, mycological eradication and overall success. Furthermore, ibrexafungerp was safe and generally well tolerated in VVC woman [10, 11].

Based on positive results of the VANISH 303/306 studies, ibrexafungerp received its first approval on 1 Jun 2021 in the US for the treatment of VVC in adult and post-menarchal pediatric females. The recommended dosage of ibrexafungerp is 300 mg twice daily for 1 day [12]. Furthermore, it was approved in the US for the prevention of recurrent vulvovaginal candidiasis (RVVC) in Nov 2022 based on another pivotal phase III clinical trial (CANDLE, NCT04029116) [12]. Ibrexafungerp is the only oral antifungal US FDA-approved treatment for VVC and reduction of RVVC.

Our study adopted a similar study design and the same dosage regimen as VANISH 303/306 study, which firstly intended to explore the efficacy and safety of ibrexafungerp in China’s VVC patients.

## Methods

### Study design and participants

This was a multicenter, randomized, double-blinded, placebo-controlled study. It aimed to assess the efficacy and safety of oral ibrexafungerp vs placebo among Chinese female patients with VVC. The pivotal study (Registry number: CTR20220918) was conducted at 31 tertiary hospitals in China (Appendix 1).

Female patients aged 18–64 (inclusive) were eligible if they were generally healthy and had a diagnosis of symptomatic VVC fulfilling the following criteria: (1) presenting at least two symptoms and/or signs that matched the vulvovaginal signs and symptoms (VSS) score (Supplementary Table 1) ≥ 2; (2) a microscopic result indicating positive *Candida* (showing hyphae/pseudohyphae/budding yeast); (3) vaginal pH ≤ 4.5 (considered as normal pH). Patients were excluded if they were pregnant, or concomitant with uncontrolled diabetes (HbA1c > 9%), other vaginal infections, or immunosuppression. A full version of the eligibility criteria is displayed in Appendix 2.

Eligible patients were randomly assigned in a 2:1 ratio to receive ibrexafungerp 300 mg (two 150-mg tablets) or matching placebo BID for 1 day. At randomization, patients were stratified by the diagnosis of diabetes mellitus (yes/no). A centralized, interactive response system was adopted for the randomization procedure. All patients, site staff and sponsor personnel were blinded to treatment assignment, except for a sponsor representative responsible for drug distribution. Placebo tablets were made indistinguishable from ibrexafungerp tablets.

The study was conducted under the guiding principles of the Declaration of Helsinki, Good Clinical Practice, and the current International Council for Harmonization of Technical Requirements for Pharmaceuticals for Human Use Guidelines. Written informed consent was obtained from all participants before the initiation of any study procedure.

### Study assessments

The study consisted of screening (day − 2 ~ − 1), baseline (day 1), test-of-cure (TOC, day 11 ± 3), and follow-up (FU, day 25 ± 4) visits. Screening and baseline visits may occur on the same day. Rating of each vulvovaginal symptom (itching, pain) of VSS score was recorded by the subject in a diary from day 1 to the TOC visit, and the procedure was done under the supervision of the investigator at screening, TOC and FU visits. Each vulvovaginal sign (congestion/edema, scratches/rhagades/erosions, secretion volume) of the VSS score was rated by the investigator based on physical examinations at screening and TOC visits, and at FU visit only when the patient was symptomatic. VSS score was calculated at each visit.

Mycological evaluation for vulvovaginal samples included fungal cultures, vaginal pH test, and microscopic examination. Vaginal pH test and microscopic examination should be performed at the local labs of study centers at screening, and at TOC and FU visits when the patient was symptomatic. Fungal cultures and susceptibility tests should be performed at central laboratories at screening and TOC visits, and at FU visit if symptomatic. Susceptibility tests were performed under Clinical and Laboratory Standards Institute (CLSI) M59 and M60 guidelines [13, 14].

The patient could return to study centers for rescue therapy if she experienced persistence, worsening, or recurrence of symptoms ideally 48–72 h after first dose of the study drug. If rescue antifungal therapy was administered before or at TOC visit, the patient would be considered a failure for the efficacy endpoints evaluated at TOC visit due to lack of efficacy. Clinical and mycological assessments should be conducted at the discretion of investigators before the prescription of rescue antifungal agents (e.g., fluconazole).

### Endpoints

The primary efficacy endpoint was the proportion of patients who reached clinical cure (VSS score = 0) at TOC visit. Main secondary efficacy endpoints included the proportion of patients with mycological eradication, overall response (achieving both clinical cure and mycological eradication), and clinical improvement at TOC visit, as well as vulvovaginal symptom resolution at FU visit. The definition of efficacy outcomes above was summarized in Supplementary Table 2. Safety endpoints focused on the incidence of treatment-emergent adverse events (TEAE), treatment-related treatment-emergent adverse events (TRAE), serious adverse events (SAE), and TEAE leading to study discontinuation.

Given an increasing emergence of NAC species and fluconazole-resistant *C. albicans*, post-hoc analyses for efficacy endpoints were conducted in patients infected by fluconazole susceptible and non-susceptible *C. albicans* and other *Candida* species.

### Statistical analyses

All analyses were performed using SAS software (version 9.4; SAS Institute Inc., Cary, NC, USA). A sample size of 258 was calculated to provide about 90% power to detect a difference between Ibrexafungerp and placebo based on Pearson’s Chi-squared test with a type I error of 5%, an assumed clinical cure rate of 56.9% for ibrexafungerp and 35.7% for placebo, and a 2:1 randomization ratio. With an estimated 30% of patients without mycological culture-confirmed infection at baseline, a sample size of 369 (Ibrexafungerp, *n* = 246; placebo, *n* = 123) was planned to be randomized. Diagnosis of diabetes mellitus was used as a stratification factor during randomization.

Categorical variables were summarized by counts and percentages. Means (± SD) were used to descriptively summarize continuous variables. A 2-sided alpha of 0.05 was used for all hypothesis tests. Safety analyses were conducted for the safety set, which included randomized patients who received ≥ 1 dose of study drug. Cochran–Mantel–Haenszel test adjusted for diagnosis of diabetes mellitus was used for efficacy analyses primarily based on the modified intent-to-treat (mITT) population, which consisted of patients in the safety population who had a positive culture for *Candida* species at baseline. Patients who received rescue antifungal treatment on or before a specific visit and patients who were missing categorical response data at specific visit were considered to be non-responders. Three sensitivity analyses were also performed: Strategy 1 performed multiple imputation (MI) for 100 times in patients lacking clinical cure data at TOC visit due to COVID-19; Strategy 2 performed Copy Reference MI for 100 times in patients lacking clinical cure data at TOC visit; Strategy 3 only included patients with collected clinical cure response data or those who received rescue antifungal treatment before or at TOC visit (considered as failure). Details of the three strategies are described in Supplementary Table 3. Subgroup analyses were performed for BMI category at screening (< 28, ≥ 28 kg/m^2^), diagnosis of diabetes mellitus, severity of infection at baseline (mild to moderate, severe), and *Candida* species at screening.

### Role of the funding source

The study was sponsored by Jiangsu Hansoh Pharmaceutical Group Co. Ltd (China). The sponsor was involved in study design, study monitoring, data collection, data analysis and interpretation, reporting of the study, and authorization of study results for publication.

## Results

### Patients

**Fig. 1:**
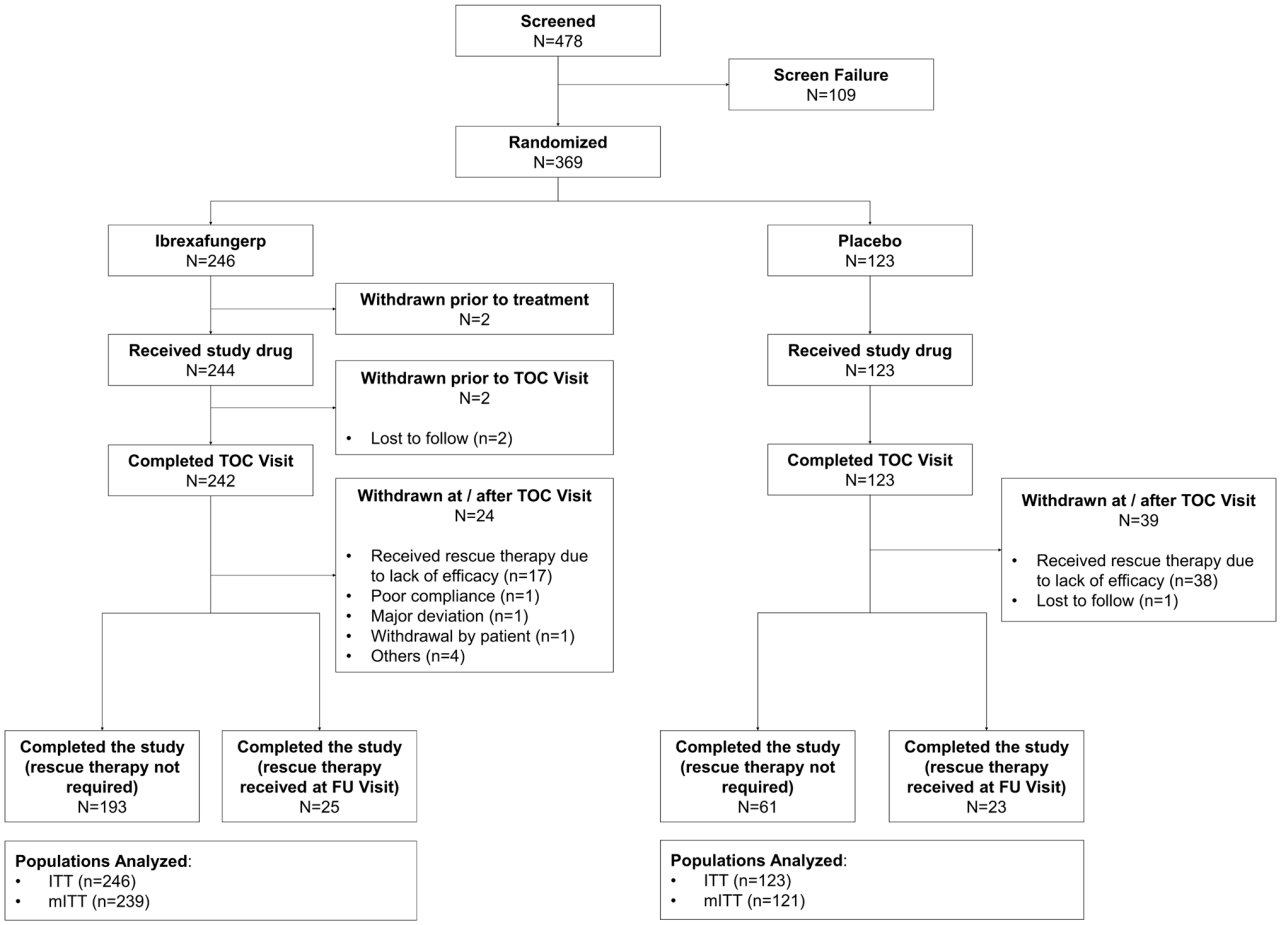
Patient disposition. *TOC* test-of-cure, *FU* follow-up, *ITT* intention-to-treat, *mITT* modified intention-to-treat

Patients were enrolled between August 2022 and February 2023 at 31 study sites in China. A total of 369 patients were randomized and assigned to either the ibrexafungerp group (*n* = 246) or placebo group (*n* = 123), among which 360 patients comprised the mITT set for primary efficacy analysis (239 for ibrexafungerp and 121 for placebo) (Fig. 1).

Demographic characteristics such as age, body mass index (BMI), proportion of menopause as well as diabetic patients between the treatment groups were comparable (Table 2). Most patients in both groups were of childbearing age, had a normal BMI range, and non-diabetic. There was also a similar pattern of VVC infection regarding severity and mean & median VSS score between the two groups at baseline. Over 80% of patients suffered mild-to-moderate VVC (VSS score < 7) and the rest suffered severe VVC (VSS score ≥ 7). The most common species cultivated at baseline was *C. albicans* (67.4% for ibrexafungerp and 62.0% for placebo), followed by *C. glabrata* and *C. krusei* (Table 2), which was consistent with the recent epidemiology of VVC in Chinese patients [6]. At screening, 20.0% (48/239) and 15.7% (19/121) patients were infected with fluconazole non-susceptible *C. albicans* in the ibrexafungerp and placebo groups, respectively (Table 1).

**Table 1:** Baseline characteristics (mITT set)

	Ibrexafungerp (*N* = 239)	Placebo (*N* = 121)
Age (years)		
Mean (SD)	32.4 (7.72)	33.5 (8.54)
Median	32.0	34.0
Min, max	18, 53	18, 58
Ethnic group, *n* (%)		
Han Chinese	208 (87.0)	105 (86.8)
Others	31 (13.0)	16 (13.2)
BMI (kg/m^2^)		
Mean (SD)	21.8 (3.14)	22.5 (3.65)
Median	21.4	21.6
Min, max	15.8, 34.5	17.0, 34.2
BMI category, *n* (%)		
< 28 kg/m^2^	228 (95.4)	110 (90.9)
≥ 28 kg/m^2^	11 (4.6)	11 (9.1)
Menopause, *n* (%)		
Yes	3 (1.3)	7 (5.8)
No	236 (98.7)	114 (94.2)
Diabetes mellitus, *n* (%)		
Yes	7 (2.9)	3 (2.5)
No	232 (97.1)	118 (97.5)
Baseline VSS score		
Mean (SD)	4.9 (2.14)	4.6 (1.77)
Median	5.0	5.0
Min, max	2, 15	2, 11
Severity based on VSS Score, *n* (%)		
Mild to moderate (< 7)	195 (81.6)	106 (87.6)
Severe (≥ 7)	44 (18.4)	15 (12.4)
Cultured *Candida* spp. at screening, *n* (%)^a^		
*C. albicans*	161 (67.4)	75 (62.0)
FLU susceptible* C. albicans*	113 (47.3)	56 (46.3)
FLU non-susceptible *C. albicans*^b^	48 (20.1)	19 (15.7)
*C. glabrata*	60 (25.1)	38 (31.4)
*C. krusei*	9 (3.8)	4 (3.3)
Other species^b^	9 (3.8)	5 (4.1)

*FLU* fluconazole, *mITT* modified intent-to-treat, *SD* standard deviation, *BMI* body mass index, *VVC* vulvovaginal candidiasis, *VSS* vulvovaginal signs and symptoms

^a^Patients may have more than 1 *Candida* species at screening and would be counted once at each species level

^b^Fluconazole non-susceptible *C. albicans* included both susceptible-dose dependent strains and resistant strains per Clinical and Laboratory Standards Institute M60 guideline

^c^Other species included *C. parapsilosis*, *C. tropicalis*, *C. metapsilosis* and *C. inconspicua*

### Efficacy endpoints

**Fig. 2:**
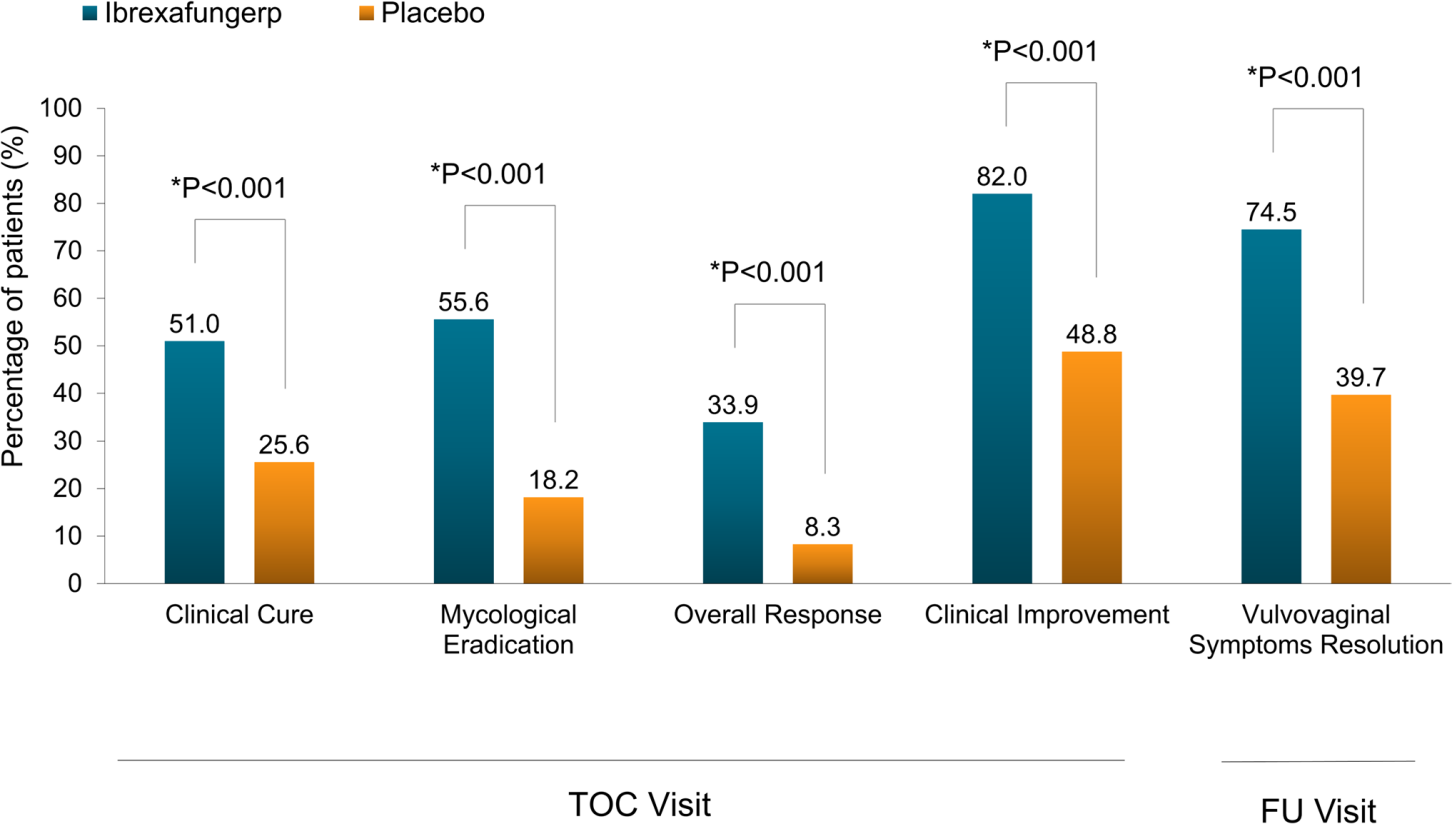
Results of primary and secondary efficacy endpoints in the mITT set. *mITT* modified intention-to-treat

As primary endpoint, the clinical cure rate at TOC visit was significantly higher with ibrexafungerp (51.0%, 122/239) than placebo (25.6%, 31/121; difference [95% CI]: 25.3% [15.31, 35.21]; *P* < 0.001) (Table 2; Fig. 2). The result of clinical cure was further supported by sensitivity analysis (Supplementary Table 3).

**Table 2:** Summary of primary and secondary endpoints (mITT Set)

Study endpoints	Ibrexafungerp, *n/N* (%)	Placebo, *n/N* (%)	Rate difference (%) (95%CI)	*P* value
Primary endpoint				
Clinical cure at TOC	51.0 (122/239)	25.6 (31/121)	25.3 (15.31, 35.21)	< 0.001
Secondary endpoints				
Mycological eradication at TOC	55.6 (133/239)	18.2 (22/121)	37.5 (28.18, 46.88)	< 0.001
Overall response at TOC	33.9 (81/239)	8.3 (10/121)	25.6 (17.84, 33.38)	< 0.001
Clinical improvement at TOC	82.0 (196/239)	48.8 (59/121)	33.2 (23.03, 43.30)	< 0.001
Complete symptom resolution at FU	74.5 (178/239)	39.7 (48/121)	34.8 (24.45, 45.11)	< 0.001

*mITT* modified intention-to-treat, *CI* confidence interval, *TOC* test-of-cure, *FU* follow-up

Ibrexafungerp demonstrated statistical superiority over placebo in all secondary endpoints (Table 2; Fig. 2). At TOC visit, mycological eradication (negative culture for *Candida* species) rate was significantly higher in the ibrexafungerp group than that of in the placebo group (55.6% [133/239] vs. 18.2% [22/121]; difference [95% CI] 38.8% [29.46, 48.21]; *P* < 0.001). Ibrexafungerp also demonstrated superiority over placebo in the percentage of patients achieving overall response (clinical cure and mycological eradication) at TOC visit (33.9% [81/239] vs. 8.3% [10/121]; difference [95% CI] 25.6% [17.84, 33.38]; *P* < 0.001), as well as patients with clinical improvement (VSS score ≤ 1) at TOC visit (82.0% [196/239] vs. 48.8% [59/121]; difference [95% CI] 33.2% [23.03, 43.30]; *P* < 0.001).

At FU visit, a larger proportion of patients in the ibrexafungerp group achieved vulvovaginal symptom resolution (74.5% [178/239] vs. 39.7% [48/121], *P* < 0.001) (Table 2; Fig. 2). Patients who missed FU visits were considered as failures. A majority of patients achieving clinical cure at TOC visit were also vulvovaginal asymptomatic at FU visit (Supplementary Table 4).

Subgroup analyses of clinical cure were performed as below. The superiority of ibrexafungerp over placebo was shown in both severe VVC patients (45.5% vs. 13.3%, *P* = 0.026) and mild-to-moderate VVC patients (52.3% vs. 27.4%, *P* < 0.001) (Table 3). For clinical cure, the efficacy of ibrexafungerp was also observed in non-obese (BMI < 28 kg/m^2^) and non-diabetic subgroups, while the efficacy could not be determined in obese and diabetic subgroups due to a limited sample size (22 obese patients and 10 diabetic patients were enrolled).

**Table 3:** Subgroup analyses for primary endpoint (mITT Set)

Subgroup	Clinical cure, *n/N* (%)		Rate difference (%), 95% CI	*P* value
	Ibrexafungerp	Placebo		
Body mass index				
< 28 kg/m^2^	120/228 (52.6)	29/110 (26.4)	26.3 (15.01, 37.09)	< 0.001
≥ 28 kg/m^2^	2/11 (18.2)	2/11 (18.2)	0.0 (− 43.56, 43.56)	1.000
Diagnosis of diabetes				
Yes	6/7 (85.7)	2/3 (66.7)	19.0 (− 47.08, 78.59)	0.490
No	116/232 (50.0)	29/118 (24.6)	25.4 (14.49, 36.06)	< 0.001
Severity of VVC				
Mild-to-moderate (VSS score < 7)	102/195 (52.3)	29/106 (27.4)	24.9 (13.27, 36.15)	< 0.001
Severe (VSS score ≥ 7)	20/44 (45.5)	2/15 (13.3)	32.1 (2.14, 58.12)	0.026
*Candida* species at screening^a^				
*C. albicans*	88/161 (54.7)	15/75 (20.0)	34.7 (21.31, 47.17)	< 0.001
*C. glabrata*	26/60 (43.3)	13/38 (34.2)	9.1 (− 11.21, 28.95)	0.369
*C. krusei*	3/9 (33.3)	2/4 (50.0)	− 16.7 (− 70.15, 43.30)	0.569

*CI* confidence interval, *VVC* vulvovaginal candidiasis, *VSS* vulvovaginal signs and symptoms

^a^Subgroup analyses would not be performed for subgroups containing fewer than ten patients

In the subgroup of those with *C. albicans*, a statistically significant result of clinical cure was found (54.7% [88/161] vs. 20.0% [15/75], *P* < 0.001), generally in line with results in the overall mITT set (Table 3). For mycological eradication, the rate difference was 55.1% (76.4% vs. 25.3%, *P* < 0.001) in those with *C. albicans* (Supplementary Table 5).

**Fig. 3:**
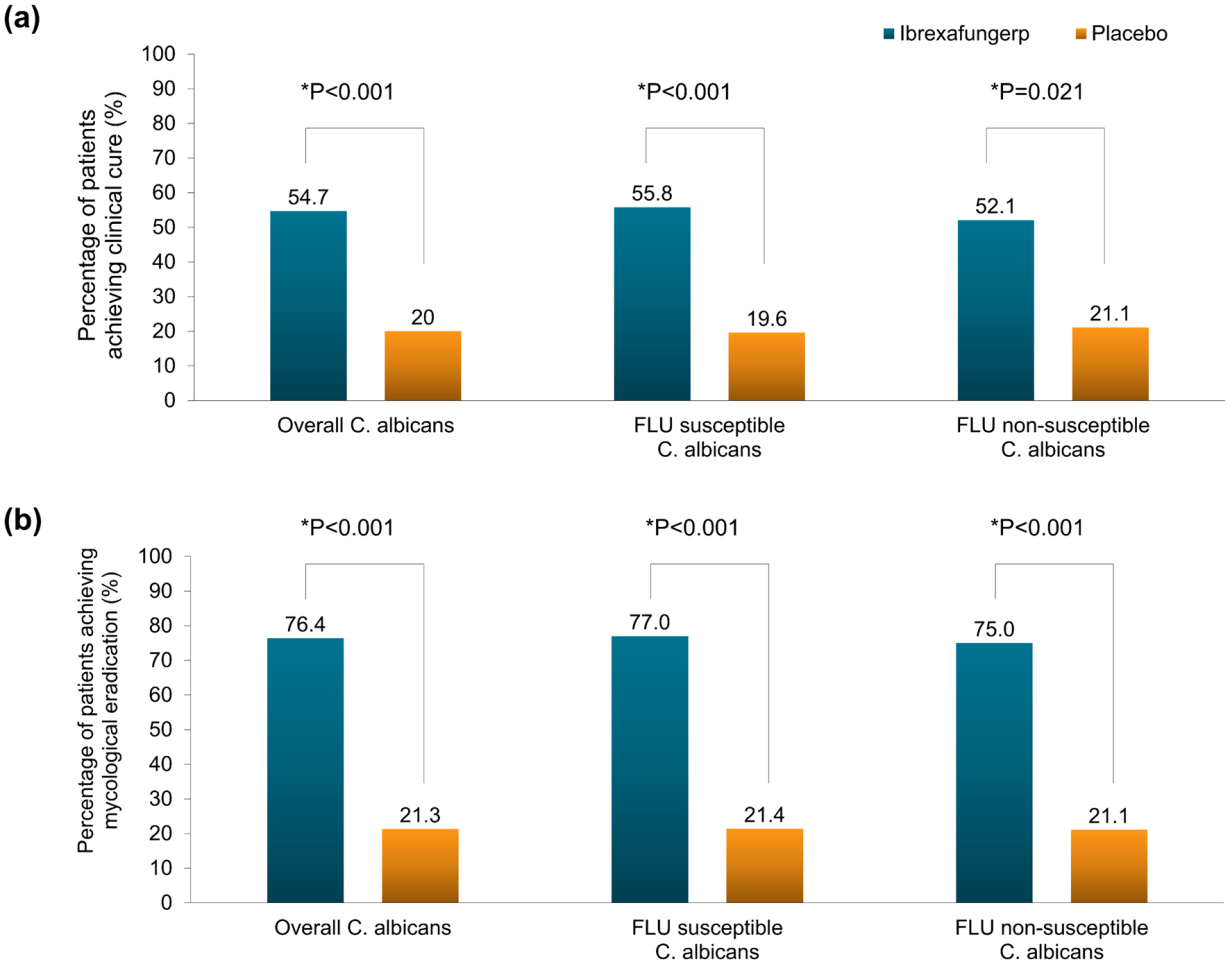
Results of clinical cure (**a**) and mycological eradication (**b**) in patients infected with *C. albicans* at test-of-cure visit

Due to an increasing proportion of fluconazole-resistant *Candida* in VVC infection in China, post-hoc analyses were explored in patients infected with fluconazole susceptible or non-susceptible *C. albicans* (per minimal inhibitory concentration [MIC]), and in patients infected with NAC. In the fluconazole non-susceptible *C. albicans* subgroups, ibrexafungerp showed superior efficacy over placebo regarding both clinical cure (52.1% vs. 21.1%, *P* = 0.021) and mycological eradication (75.0% vs. 21.1%, *P* < 0.001) (Supplementary Table 5; Supplementary Table 6). In the ibrexafungerp group, there were similar efficacy results between fluconazole non-susceptible *C. albicans* subgroup and overall *C. albicans* subgroup (Fig. 3A, B). There was also a trend that patients infected with NAC had a higher clinical cure rate in the ibrexafungerp group (Supplementary Table 6). While, the mycological eradication rate did not differ in NAC subgroups (Supplementary Table 5). The percentage of patients who achieved clinical cure but remained positive for *Candida* culture at TOC visit was also summarized by species infected at screening (Supplementary Table 7).

### Safety

Overall, ibrexafungerp was well tolerated. At least one TEAE was reported by 63.1% and 42.3% of patients receiving ibrexafungerp and placebo, respectively (Table 4). All except one TEAEs were mild to moderate in severity. As for TRAE, 54.1% of patients receiving ibrexafungerp reported one or more TRAEs compared to 17.1% receiving placebo (Supplementary Table 8). By system organ class (SOC), TEAEs of gastrointestinal disorders were most frequently reported for ibrexafungerp (50.8% [124/244]), whereas infections and infestations were mostly reported for placebo (17.1% [21/123]). By preferred term (PT), the most common TEAE in the ibrexafungerp group was diarrhea (43.0%), followed by nausea (9.0%) and abdominal pain (4.1%). There existed consistency between TRAEs and TEAEs regarding the incidence and severity of SOC and PT.

**Table 4:** Summary of treatment-emergent adverse events (TEAEs) reported in ≥ 2% of patients (safety set) ^a^

System organ class Preferred term	Ibrexafungerp (*N* = 244) *n* (%)	Placebo (*N* = 123) *n* (%)
Patient with ≥ 1 TEAE	154 (63.1)	52 (42.3)
Gastrointestinal disorders	124 (50.8)	13 (10.6)
Diarrhea	105 (43.0)	6 (4.9)
Nausea	22 (9.0)	0
Abdominal pain	10 (4.1)	2 (1.6)
Upper abdominal pain	7 (2.9)	0
Dry mouth	2 (0.8)	3 (2.4)
Gastroesophageal reflux disease	0	3 (2.4)
Infections and infestations	33 (13.5)	21 (17.1)
Upper respiratory tract infection	9 (3.7)	3 (2.4)
Bacterial vulvovaginitis	7 (2.9)	10 (8.1)
Vaginal infection	4 (1.6)	5 (4.1)
Bacterial vaginosis	3 (1.2)	5 (4.1)
Investigations	18 (7.4)	10 (7.1)
Positive SARS-CoV-2 test	8 (3.3)	4 (3.3)
Nervous system disorders	16 (6.6)	11 (8.9)
Dizziness	12 (4.9)	7 (5.7)
Headache	3 (1.2)	3 (2.4)

^a^At each level of patient summarization, a patient is counted once if the patient reported ≥ 1 events

No TEAE led to study discontinuation. Two SAEs were separately reported by two patients in the placebo group, which were “papillary thyroid carcinoma” and “colorectal polyp, Barrett esophagus, esophageal papilloma, duodenal polyp”, respectively. Neither of the SAEs was treatment-related SAE. One pregnancy was reported in the ibrexafungerp group during the study, and the outcome was elective abortion.

## Discussion

This was the first clinical trial to evaluate the efficacy and safety of ibrexafungerp in Chinese VVC patients. Our study demonstrated that ibrexafungerp was efficacious and well tolerated in Chinese VVC patients under the conditions of this study. The clinical cure (absence of vulvovaginal symptoms and signs) rates with ibrexafungerp vs. placebo in our study (51.0% vs. 25.6%, respectively) were similar to that of VANISH 303 study (50.5% vs. 28.6%) as well as US patients in VANISH 306 study (54.5% vs. 27.8%) [10, 11]. In the present study, ibrexafungerp demonstrated reproducible statistical superiority over placebo for VVC treatment in Chinese patients, in consistency with the result of similarly designed VANISH 303/306 study.

Our study revealed the sustained efficacy of ibrexafungerp. In the ibrexafungerp group, 80.3% of patients who achieved clinical cure at TOC visit (day 11 ± 3) remained asymptomatic at FU visit (day 25 ± 4). Whereas, several published studies showed that fluconazole under various regimens reported an 11%-20% decrease in sustained response from day 7–14 to day 28–35[15–17]. It may be attributed to the fungicidal activity of ibrexafungerp in comparison to the fungistatic activity of fluconazole [18, 19]. Therefore, it indicated that ibrexafungerp was superior to fluconazole in sustained therapeutic effect.

Based on the susceptibility tests for fluconazole against *Candida* species obtained at screening, our study also explored the efficacy of ibrexafungerp in both patients with fluconazole-susceptible and patients with non-susceptible *C. albicans*. In the ibrexafungerp group, it was observed a similar clinical cure rate between patients with fluconazole-susceptible and non-susceptible *C. albicans* (55.8% vs. 52.1%). Moreover, in the ibrexafungerp group, patients infected with fluconazole susceptible and non-susceptible *C. albicans* both had a high-mycological eradication rate (77.0% and 75%). The clinical benefits of ibrexafungerp for patients with fluconazole non-susceptible *C. albicans* were consistent with a preclinical study. In the preclinical study, MIC of the *Candida* isolates against ibrexafungerp was determined per broth microdilution method published by CLSI. A total of 178 *Candida* were tested, including 44 *Candida* isolates with known genotypic (*FKS1* or *FKS2* mutations), phenotypic, or clinical resistance to echinocandins. Ibrexafungerp MICs were low (≤ 0.5 μg/ml) for azole-resistant *C. albicans*, *C. parapsilosis,* and *C. tropicalis* isolates, which demonstrated in vitro fungicidal activity of ibrexafungerp against azole-resistant *Candida* species [20]. Nowadays, fluconazole-resistant *C. albicans* has been a growing and perplexing problem for treating VVC. One up-to-date literature published in 2022 collected 2000 *Candida* isolates from VVC patients in 23 hospitals to explore the sensitivity of *Candida* species to common antifungals in China. The result showed that the resistance rate for vulvovaginal *C. albicans* was significantly higher than that of non-*C. albicans* (73.41% [715/974] vs. 50.88% [115/226], *P* < 0.001). [6]. Based on the positive results of our study, ibrexafungerp would probably be an alternative option for treatment of patients with fluconazole non-susceptible *C. albicans* in China.

Among patients with NAC in the ibrexafungerp group, the clinical cure rate was 43.6%, while the mycological eradication rate was just 12.8% at TOC visit. Of these, 67.6% of patients with positive culture for NAC were asymptomatic. The inconsistency between clinical cure and mycological eradication rate may be explained by the *Candida* colonization in vagina. A previous study of Kennedy et al. showed that at least 50% of women with positive cultures for NAC might be minimally symptomatic or had no symptoms, while 80–85% of patients with positive cultures for *C. albicans* would be likely to have symptoms [21], which was consistent with our study. Moreover, clinical practice guidelines recommended that asymptomatic patients with mere mycological persistence required no further therapy [3, 22].

According to pharmaceutical industry guidance issued by the Food and Drug Administration in 2019, our study chose placebo rather than fluconazole as a comparator using a superiority design and adopted clinical cure (i.e., the complete resolution of signs and symptoms of VVC without need for further antifungal treatment before or at TOC visit) as the primary endpoint. Nevertheless, the efficacy of single-dose fluconazole and single-day ibrexafungerp was compared in a US phase II study (DOVE), in which the clinical cure rate in ibrexafungerp group was similar with that of fluconazole group on day 10 (51.9% vs. 58.3%) but higher than that of fluconazole group on day 25 (70.4% vs. 50.0%)[23]. Moreover, a randomized, double-blind phase III trial of a new antifungal agent in China adopted the same definition of clinical cure (VSS score = 0) and mITT population. The study found that in the fluconazole group, the percentage of subjects reaching clinical cure on day 14 in the mITT population and subgroup of *C. albicans* was 50.31% and 53.72%, respectively, which is similar to those of ibrexafungerp in our study (51.0% for mITT population; 55.8% for a subgroup of *C. albicans* on day 11 ± 3) [24].

Ibrexafungerp was well tolerable in China’s VVC patients when administered as a 300 mg oral tablet BID for 1 day. Although, a higher percentage of patients receiving ibrexafungerp (54.1%) experienced at least one TRAE compared with those receiving placebo (17.1%), most TRAEs were mild to moderate and recovered without any intervention. Consistent with VANISH 303/306 study, the most common TRAEs were gastrointestinal disorders. The incidence of gastrointestinal disorders in Chinese patients was similar to that of U.S. patients in VANISH 303/306 study (50.8% vs. 42.9%). Chinese patients experienced a higher incidence of diarrhea than U.S. patients (43.0% vs. 23.9%), but fewer nausea and abdominal pain (9.0% each) than US patients (16.8% and 14.8%, respectively) [25]. It was speculated that the differences in dietary habits between Chinese and US patients contributed to the various gastrointestinal reactions. All three clinical trials demonstrated the good safety of ibrexafungerp.

While, some limitations existed in our study the clinical efficacy of ibrexafungerp in obese or diabetic patients could not be determined in the subgroup analyses due to the small sample size. Therefore, future research on ibrexafungerp was warranted, especially its effectiveness in specific patients.

## Conclusion

In conclusion, ibrexafungerp is a novel, oral antifungal with a statistically superior efficacy to placebo. Moreover, ibrexafungerp was well tolerated in Chinese patients and most TRAEs were gastrointestinal disorders that were mild to moderate in nature. Given the significant clinical benefits and good safety of ibrexafungerp, it would probably be a new option for Chinese VVC patients in the future.

## Supplementary Information

Below is the link to the electronic supplementary material.

Supplementary file1 (DOCX 37 KB)

## Data Availability

The source data that support the findings of this study are available from Jiangsu Hansoh Pharmaceutical Group Co., Ltd. Restrictions apply to the availability of these data, which were used under license for this study. Data are available from the corresponding author (Qinping Liao) with the permission of Jiangsu Hansoh Pharmaceutical Group Co., Ltd.
