# Supplementary material for: Efficacy and safety of oral ibrexafungerp in Chinese patients with vulvovaginal candidiasis: a phase III, randomized, double-blind study
Source: Infection. 2024 Apr 3;52(5):1787–97. doi: 10.1007/s15010-024-02233-w (PMC11499449; doi:10.1007/s15010-024-02233-w)
Supplement: Supplementary file 1 — Supplementary file1 (DOCX 37 KB) [file 15010_2024_2233_MOESM1_ESM.docx]

Supplementary Materials

**Appendix 1 Principal Investigators and Study Sites**

| Investigator Name | Facility Name | Address |
| --- | --- | --- |
| Qinping Liao | Beijing Tsinghua Changgung Hospital | 168 Litang Road, Changping District, Beijing |
| Wenying Wang | The First Affiliated Hospital of Xi'an Medical College | No. 48 Fenghao West Road, Lianhu District, Xi'an City, Shaanxi Province |
| Jingjing Li | Liuzhou Maternity and Child Healthcare Hospital | No. 50 Yingshan Street, Chengzhong District, Liuzhou city, Guangxi Zhuang Autonomous Region |
| Ruifang An | The First Affiliated Hospital of Xi'an Jiaotong University | 277 Yanta West Road, Yanta District, Xi 'an City, Shaanxi Province |
| Lihong Chen | Shaanxi Provincial People's Hospital | 256 Youyi West Road, Xi'an City, Shaanxi Province |
| Jiajing Lin | Liuzhou Worker's Hospital | 156 Heping Road, Liunan District, Liuzhou City, Guangxi Zhuang Autonomous Region |
| Dabao Xu | THE THIRD XIANGYA HOSPITAL OF CENTRAL SOUTH UNIVERSITY | 138 Tongzipo Road, Hexi Yuelu District, Changsha City, Hunan Province |
| Jin Qiu | Shanghai Tongren Hospital | Tongren Hospital, 111 Xianxia Road, Changning District, Shanghai |
| Weihua Song | Women & Children's Health Care Hospital of Linyi | No. 1 Qinghe South Road, Luozhuang District, Linyi |
| Mijiti Patiman | UNIVERSITY OF CHINESE ACADEMY OF SCIENCES SHENZHEN HOSPITAL | No. 4253 Songbai Road, Matan Street, Guangming District, Shenzhen City, Guangdong Province |
| Hongjie Ruan | Nanjing Women and Children's Healthcare Hospital | No. 123, Tianfei Lane, Mochou Road, Nanjing, Jiangsu, China |
| Gang Wang | Sichuan Maternal and Child Health Hospital | 290 Shayan West 2nd Street, Wuhou District, Chengdu City, Sichuan Province |
| Fengxia Xue | Tianjin Medical University General Hospital | No. 154 Anshan Road, Heping District, Tianjin |
| Xu Wang | Tonghua Central Hospital | 176 Xinguang Road, Jianshe Street, Tonghua City |
| Xiaowan Luo | Zhongshan Women and Children's Hospital | No. 6 Chenggui Road, East District, Zhongshan City, Guangdong Province |
| Qi Ruan | Shunde Women and Children's Hospital of Guangdong Medical University | No. 3, Health Care Road, Daliang Street, Shunde District, Foshan City, Guangdong Province |
| Ling Shi | The Second Affiliated Hospital of Liaoning University of Traditional Chinese Medicine | No. 60, Huanghe North Street, Huanggu District, Shenyang City |
| Chun Zhang | THE CENTRAL HOSPITAL OF WUHAN | 26 Shengli Street, Jiang'an District, Wuhan City, Hubei Province |
| Lina Hu | THE SECOND AFFILIATED HOSPITAL OF CHONGQING MEDICAL UNIVERSITY | No. 74, Linjiang Road, Yuzhong District, Chongqing |
| Shijin Wang | The First Affiliated Hospital of Xinxiang Medical University | No. 88, Jiankang Road, Weihui City, Xinxiang, Henan province |
| Hong Shi | Dalian Medical University Affiliated First Hospital | 222 Zhongshan Road, Dalian City |
| Xiaoli Wang | HAINAN WOMEN AND CHILDREN'S MEDICAL CENTER | No. 15, Longkun South Road, Haikou City, Hainan |
| Songling Zhang | The First Hospital of Jilin University | No. 1 Xinmin Street, Changchun City |
| Yingxiong Li | Guangzhou Panyu Central Hospital | No. 8, Fuyu East Road, Qiaonan Street, Panyu District, Guangzhou |
| Jing Lu | Urumqi Maternal and Child Health Hospital | 344 Jiefang South Road, Tianshan District, Urumqi, Xinjiang Uygur Autonomous Region |
| Baojin Wang | THE THIRD AFFILIATED HOSPITAL OF ZHENGZHOU UNIVERSITY | No. 7, Kangfuqian Road, Erqi District, Zhengzhou City, Henan Province |
| Hongyan Xu | Yuebei People's Hospital | 133 Huimin South Road, Wujiang District, Shaoguan City, Guangdong Province |
| Hong Ye | Yichang Central People's Hospital | No. 183 Yiling Road, Yichang City, Hubei Province |
| Bei Zhang | Xuzhou Central Hospital | No. 199, Jiefang South Road, Xuzhou, Jiangsu |
| Chunlian Zhang | Taihe Hospital, Affiliated Hospital of Hubei University of Medicine | 30 South Renmin Road, Shiyan, Hube |
| Sumin Qian | Cangzhou Central Hospital | 16 Xinhua West Road, Yunhe District, Cangzhou City, Hebei Province |

**Appendix 2 Inclusion and Exclusion Criteria**

Subjects must fulfill all of the inclusion criteria and none of the exclusion criteria to be eligible for study admission:

**Inclusion Criteria:**

1. Subject should be fully aware of the content, procedure as well as possible AE of the study and should sign written ICF. Subject is a female subject aged 18 to 65 years (inclusive).
2. Subject has a diagnosis of symptomatic AVVC that meets the following criteria:
   - Minimum composite vulvovaginal signs and symptoms (VSS) score of ≥2 with at least 2 signs or symptoms at Screening
   - Positive microscopic examination via a vaginal sample collected at Screening revealing yeast forms (hyphae/pseudohyphae) or budding yeasts
   - Normal vaginal pH (≤4.5)
3. Subject is able to take oral tablets.
4. Subject is in general health based on medical history, demographic data, physical examination, vital sign measurements (blood pressure, pulse, breath, body temperature), SpO_2_, safety laboratory tests (hematology, coagulation function, blood biochemistry, urine routine, etc.) and electrocardiogram (ECG) performed at the Screening visit and/or prior to administration of the initial dose of study drug.
5. Subject agrees to keep abstinence between Screening and TOC visit.
6. Subject of reproductive potential has a negative result of pregnancy test at Screening.
7. Subject is not pregnant or lactating and is highly unlikely to become pregnant since she meets at least one of the following criteria:

- Subject is a female subject who is not of reproductive potential and is eligible without requiring the use of contraception. A female subject who is not of reproductive potential is defined as one who:
- has reached natural menopause (defined as 6 months of spontaneous amenorrhea with serum follicle-stimulating hormone levels in the postmenopausal range as determined by the local laboratory, or 12 months of spontaneous amenorrhea); spontaneous amenorrhea does not include cases for which there is an underlying disease that causes amenorrhea (e.g. anorexia nervosa).
- has undergone bilateral oophorectomy and/or hysterectomy or
- is 3 months post bilateral tubal ligation.
- Subject is a female subject who is of reproductive potential and is using an effective contraceptive method including intrauterine device, hormonal contraceptives (i.e., vaginal ring, implant, oral, injectable or patch) for at least 30 days before baseline, and agrees to continue using the contraceptive method through at least 10 days after the completion of study therapy. Vasectomy in the male partner is also an acceptable contraceptive method as long as performed at least 3 months prior to Screening.

Subjects must refrain from using any topical vaginal contraceptives as these may have an impact on the signs and symptoms of VVC.

**Exclusion Criteria:**

1. Subject has any vaginal condition other than VVC that may interfere with the diagnosis or evaluation of response to therapy, such as suspected or confirmed concurrent causes of vulvovaginitis and/or cervicitis including bacterial vaginosis, *Trichomonas* vaginitis, symptomatic human papillomavirus or other mixed infections.
2. Subject has active menstruation at Screening visit (Screening can be postponed if necessary).
3. Subject has uncontrolled diabetes mellitus (HbA1c > 9%).
4. Subject has a history of or an active cervical/vaginal cancer.
5. Subject requires treatment with the prohibited medications (including prescription and over-the-counter medications, supplements, and herbal products) during the following timeframes:

- Systemic and/or topical (vaginal) antifungal treatment, including prescription or over-the-counter products, within 28 days prior to enrollment if administered for the treatment of VVC and during the study for all cases
- Select strong CYP3A4/5 inhibitors and CYP3A4/5 inducers during the 7 days prior to enrollment and during study treatment until the TOC visit
- Select P-gp substrates during the 48 hours prior to enrollment or during study treatment.

1. Subject has a known hypersensitivity to any of the components of the formulation.
2. Subject has a known human immunodeficiency virus infection and/or is receiving chemotherapy or has an illness that is serious enough to induce an immune deficiency.
3. Subject has had any major illness (acute myocardial infarction, cerebral infarction, malignancy, etc.) within 30 days before Screening.
4. Subject has participated in any other investigational study within at least 30 days (or 5 half-lives of the investigational product) before signing the ICF.
5. Subject has received prior treatment with HS-10366 in a previous trial.
6. Subject has any other condition or laboratory abnormality that would put the subject at unacceptable risk for participation in the study or may interfere with the compliance or the assessments included in the study.
7. Subject is not suitable to participate in the study in the judgment of the investigator.

**Supplementary Table 1 Vulvovaginal Signs and Symptoms (VSS) Score**

**Part 1: Symptoms**

**To be rated by the patient in Patient Diary and at each visit under the supervision of the investigator**

| Score | Pruritus | Pain |
| --- | --- | --- |
| 0 | Absent | Absent |
| 1 | Mild | Mild |
| 2 | Moderate | Moderate |
| 3 | Severe | Severe |

**Part 2: Signs**

**To be rated by the investigator during vulvovaginal examination at each visit**

| Score | Congestion/edema | Scratches/rhagades/erosions | Secretion volume |
| --- | --- | --- | --- |
| 0 | Absent | Absent | Absent |
| 1 | Mild | - | Mild |
| 2 | Moderate | - | Moderate |
| 3 | Severe | Present | Severe |

VSS score is the composite score of all the items of symptoms and signs.

**Supplementary Table 2 Definition of Efficacy Endpoints**

| Endpoint | Description |
| --- | --- |
| Clinical cure | Complete resolution of vulvovaginal signs and symptoms prior to or at TOC visit and no antifungal agent used/required. VSS score = 0. |
| Mycological eradication | Negative culture for *Candida* species at TOC visit and no antifungal agent used/required. |
| Overall success | Clinical cure and mycological eradication at TOC visit. |
| Clinical improvement | Partial or complete resolution of vulvovaginal signs and symptoms prior to or at TOC visit and no antifungal agent used/required. VSS score ≤1. |
| Vulvovaginal symptom resolution at FU | Complete resolution of vulvovaginal symptoms (i.e. itching and pain) prior to or at FU visit and no antifungal agent used/required. Symptom of VSS score = 0. |

Abbreviations: TOC, test-of-cure; VSS, vulvovaginal signs and symptoms; FU, follow-up.

**Supplementary Table 3 Sensitivity Analyses for Primary Endpoint (mITT Set)**

| **Sensitivity**  **Analyses** | **Clinical cure, n/N (%)** | | | **Rate differences (%)**  **95%CI** | **P value** |
| --- | --- | --- | --- | --- | --- |
|  | **Ibrexafungerp** | **Placebo** | |  |  |
| Strategy 1^a^ | 122.5/239 (51.3)^d^ | | 31.0/121 (25.6)^d^ | 25.5 (15.51, 35.43) | <0.001 |
| Strategy 2^b^ | 122.4/239 (51.2)^d^ | | 31.0/121 (25.6)^d^ | 25.4 (15.48, 35.41) | <0.001 |
| Strategy 3^c^ | 122/237 (51.5) | | 31/121 (25.6) | 25.7 (15.71, 35.65) | <0.001 |

The primary analysis for clinical cure regarded patients with missing data as non-responders after addressing intercurrent events.

^a^. Strategy 1 performed MI for 100 times in patients lacking clinical cure data at TOC visit due to COVID-19. Variables in the MI model consisted of treatment group, diagnosis of diabetes (yes or no) and VVC score at baseline. Patients lacking clinical cure data at TOC visit due to other reasons were considered as failure.

^b^. Strategy 2 performed Copy Reference MI for 100 times in patients lacking clinical cure data at TOC visit. The imputation model used data of the placebo group and included diagnosis of diabetes (yes or no) and VVC score at baseline as covariates.

^c^. Strategy 3 included patients with collected clinical cure response data or those who received rescue anti-fungal treatment prior to or at TOC visit (considered as failure).

^d^. Number and percentage of patients were the mean values after MI for 100 times.

Abbreviations: CI, confidence interval; MI, multiple imputation; n/N, number of patients achieving clinical cure versus number of patients in the subgroup.

**Supplementary Table 4 Percentage of Asymptomatic/Symptomatic VVC Patients at FU Visit Who Achieved Clinical Cure at TOC Visit (mITT Set)**

|  | **Ibrexafungerp**  **(N=122)** | **Placebo**  **(N=31)** |
| --- | --- | --- |
| Asymptomatic patients, n(%) | 98 (80.3) | 22 (71.0) |
| Symptomatic patients, n(%) | 24 (19.7) | 9 (29.0) |

Abbreviations: mITT, modified intention-to-treat; TOC, test-of-cure; VVC, vulvovaginal candidiasis.

**Supplementary Table 5 Statistical Analyses for Mycological Eradication by *Candida* Species at Screening (mITT Set)**

| ***Candida* species at screening** | **Mycological eradication, n/N (%)** | | | **Rate difference (%), 95%CI** | **P value** |
| --- | --- | --- | --- | --- | --- |
|  | **Ibrexafungerp** | **Placebo** | |  |  |
| ***C. albicans*** | | | | | |
| Overall | 123/161 (76.4) | | 16/75 (21.3) | 55.1 (42.65, 66.21) | <0.001 |
| Fluconazole susceptible (post-hoc) | 87/113 (77.0) | | 12/56 (21.4) | 55.6 (41.02, 68.66) | <0.001 |
| Fluconazole non-susceptible (post-hoc) ^a^ | 36/48 (75.0) | | 4/19 (21.1) | 54.0 (28.40, 75.55) | <0.001 |
| **NAC** | | | | | |
| Overall (post-hoc) | 34/78 (43.6) | | 16/47 (34.0) | 9.0 (-7.79, 25.79) | 0.304 |

^a^. Fluconazole non-susceptible *C. albicans* included both susceptible-dose dependent strains and resistant strains per Clinical and Laboratory Standards Institute M60 guideline.

Abbreviations: CI, confidence interval; NAC, non-*albicans Candida*.

**Supplementary Table 6 Statistical Analyses for Clinical Cure by *Candida* Species at Screening (mITT Set)**

| ***Candida* species at screening** | **Clinical cure, n/N (%)** | | | **Rate difference (%), 95%CI** | **P value** |
| --- | --- | --- | --- | --- | --- |
|  | **Ibrexafungerp** | **Placebo** | |  |  |
| ***C. albicans*** | | | | | |
| Overall | 88/161 (54.7) | | 15/75 (20.0) | 34.7 (21.31, 47.17) | <0.001 |
| Fluconazole susceptible (post-hoc) | 63/113 (55.8) | | 11/56 (19.6) | 36.1 (20.85, 50.70) | <0.001 |
| Fluconazole non-susceptible (post-hoc) ^a^ | 25/48 (52.1) | | 4/19 (21.1) | 31.0 (4.74, 55.07) | 0.021 |
| **NAC** | | | | | |
| Overall (post-hoc) | 10/78 (12.8) | | 6/47 (12.8) | 0.0 (-12.23, 12.27) | 0.998 |

^a^. Fluconazole non-susceptible *C. albicans* included both susceptible-dose dependent strains and resistant strains per Clinical and Laboratory Standards Institute M60 guideline.

Abbreviations: CI, confidence interval; NAC, non-*albicans Candida*.

**Supplementary Table 7 Percentage of Patients Who Achieved Clinical Cure but with Mycological Persistence at TOC Visit (Post hoc Analysis)**

|  | **Ibrexafungerp**  **n/N (%)** | **Placebo**  **n/N (%)** |
| --- | --- | --- |
| Overall mITT set | 71/106 (67.0) | 45/99 (45.5) |
| *C. albicans* subgroup | 25/38 (65.8) | 23/59 (39.0) |
| NAC subgroup | 46/68 (67.6) | 22/40 (55.0) |

Abbreviations: mITT, modified intention-to-treat; NAC, non-*albicans Candida*; TOC, test-of-cure.

**Supplementary Table 8 Summary of TRAEs Reported in ≥2% of Patients (Safety Set) ^a^**

| SOC/PT ^b^  Severity | Ibrexafungerp (N=244)  n (%) | Placebo (N=123)  n (%) |
| --- | --- | --- |
| Patient with ≥1 TRAE | **132 (54.1)** | **21 (17.1)** |
| Mild | 124 (50.8) | 21 (17.1) |
| Moderate | 8 (3.3) | 0 |
| Gastrointestinal disorders | **120 (49.2)** | **12 (9.8)** |
| Mild | 113 (46.3) | 12 (9.8) |
| Moderate | 7 (2.9) | 0 |
| Diarrhea | 105 (43.0) | 6 (4.9) |
| Mild | 100 (41.0) | 6 (4.9) |
| Moderate | 5 (2.0) | 0 |
| Nausea | 20 (8.2) | 0 |
| Mild | 20 (8.2) | 0 |
| Abdominal pain | 9 (3.7) | 2 (1.6) |
| Mild | 8 (3.3) | 2 (1.6) |
| Moderate | 1 (0.4) | 0 |
| Upper abdominal pain | 6 (2.5) | 0 |
| Mild | 4 (1.6) | 0 |
| Moderate | 2 (0.8) | 0 |
| Nervous system disorders | **14 (5.7)** | **8 (6.5)** |
| Mild | 13 (5.3) | 8 (6.5) |
| Moderate | 1 (0.4) | 0 |
| Dizziness | 10 (4.1) | 7 (5.7) |
| Mild | 9 (3.7) | 7 (5.7) |
| Moderate | 1 (0.4) | 0 |
| Infections and infestations | **8 (3.3)** | **0** |
| Mild | 8 (3.3) | 0 |
| Bacterial vulvovaginitis | 6 (2.5) | 0 |
| Mild | 6 (2.5) | 0 |

^a^ At each combination level of severity and causality, a patient is counted once if the patient reported ≥1 events.

^b^ SOC and PT were shown in bold and regular, respectively.

Abbreviations: SOC, system organ class; PT, preferred term; TRAE, treatment-related adverse event.
